# Pharmacogenetics of Drug Metabolism: The Role of Gene Polymorphism in the Regulation of Doxorubicin Safety and Efficacy

**DOI:** 10.3390/cancers14215436

**Published:** 2022-11-04

**Authors:** Alina A. Bagdasaryan, Vladimir N. Chubarev, Elena A. Smolyarchuk, Vladimir N. Drozdov, Ivan I. Krasnyuk, Junqi Liu, Ruitai Fan, Edmund Tse, Evgenia V. Shikh, Olga A. Sukocheva

**Affiliations:** 1 Federal State Autonomous Educational Institution of Higher Education, I.M. Sechenov First Moscow State Medical University of the Ministry of Healthcare of the Russian Federation (Sechenovskiy University), 8-2 Trubetskaya Str., 119991 Moscow, Russia; 2 The First Affiliated Hospital of Zhengzhou University, Zhengzhou 450000, China; 3 Department of Hepatology, Royal Adelaide Hospital, Adelaide, SA 5000, Australia; 4 College of Nursing and Health Sciences, Flinders University, Bedford Park, SA 5042, Australia

**Keywords:** breast cancer, doxorubicin, drug toxicity, pharmacogenetics, gene polymorphism, cytochrome P450, MDR1 protein, pharmacokinetics

## Abstract

**Simple Summary:**

The effectiveness and safety of the anti-cancer agent doxorubicin (anthracycline group medicine) depend on the metabolism and retention of the drug in the human organism. Polymorphism of cytochrome p450 (CYP)-encoding genes and detoxifying enzymes such as CYP3A4 and CYP2D6 were found responsible for variations in the doxorubicin metabolism. Transmembrane transporters such as p-glycoproteins were reported to be involved in cancer tissue retention of doxorubicin. ATP-binding cassette (ABC) family members, including ABCB1 transporters (also known as Multi-Drug Resistance 1 (MDR1)) proteins, were determined to pump out doxorubicin from breast cancer cells, therefore reducing the drug effectiveness. This study critically discusses the latest data about the role of CYP3A4, CYP2D6, and ABCB1 gene polymorphism in the regulation of doxorubicin’s effects in breast cancer patients. The assessment of genetic differences in the expression of doxorubicin metabolizing and transporting enzymes should be explored for the development of personalized medical treatment of breast cancer patients.

**Abstract:**

Breast cancer (BC) is the prevailing malignancy and major cause of cancer-related death in females. Doxorubicin is a part of BC neoadjuvant and adjuvant chemotherapy regimens. The administration of anthracycline derivates, such as doxorubicin, may cause several side effects, including hematological disfunction, gastrointestinal toxicity, hepatotoxicity, nephrotoxicity, and cardiotoxicity. Cardiotoxicity is a major adverse reaction to anthracyclines, and it may vary depending on individual differences in doxorubicin pharmacokinetics. Determination of specific polymorphisms of genes that can alter doxorubicin metabolism was shown to reduce the risk of adverse reactions and improve the safety and efficacy of doxorubicin. Genes which encode cytochrome P450 enzymes (CYP3A4 and CYP2D6), p-glycoproteins (ATP-binding cassette (ABC) family members such as Multi-Drug Resistance 1 (MDR1) protein), and other detoxifying enzymes were shown to control the metabolism and pharmacokinetics of doxorubicin. The effectiveness of doxorubicin is defined by the polymorphism of cytochrome p450 and p-glycoprotein-encoding genes. This study critically discusses the latest data about the role of gene polymorphisms in the regulation of doxorubicin’s anti-BC effects. The correlation of genetic differences with the efficacy and safety of doxorubicin may provide insights for the development of personalized medical treatment for BC patients.

## 1. Introduction

According to the World Health Organization (WHO), oncological diseases represent a global health burden with exceedingly high death rates [1,2,3]. Breast cancer (BC) is the most common type of malignant tumor diagnosed in women. Annually, 1.67 million new cases are diagnosed worldwide, representing a quarter of all cancer types [2]. In 2020, BC caused 685 thousand deaths worldwide [3]. Distant metastases, a sign of poor prognosis, are found in 20–30% of women with BC. BC incidence and mortality rates are high in many Western countries, including the United Kingdom, Canada, and the United States, where BC incidence was found to be 129.2, 99.7, and 123.1 per 100,000 women, respectively [3,4,5]. BC risk factors include elderly age, obesity, excessive alcohol consumption, smoking, radioactive exposure, and hormone replacement therapy [1,4]. However, BC onset can be also caused by inherited or acquired mutations in specific genes, such as BRCA1 and BRCA2 (breast cancer gene 1 and 2) [3,6,7]. Reflecting the heterogeneity of this malignancy, most BCs are sporadic and occur in patients who have no family history of oncological diseases. The impact of genetic polymorphism is much harder to estimate, as nearly every BC patient has a unique genetic profile.

Tumors with mutated BRCA1 are more likely to have a basal-like phenotype and do not express estrogen and/or progesterone receptors (ER, PR) or human epidermal growth factor receptor 2 (HER2). This type of BC (ER/PR/HER2-negative) is often defined as triple-negative and represents the most aggressive disease, with less promising treatment options [8,9]. Other major genes associated with higher BC incidence include phosphatase and tensin homolog (PTEN) [10], tumor-suppressor protein TP53 [11], CDH1 (which encodes epithelial cadherin or E-cadherin (E-cad) protein) [12], and serine/threonine kinase 11 (STK11) [13,14,15]. Heterogeneity of BCs is targeted by complex treatment approaches, using neoadjuvant therapy, adjuvant therapy, surgery, radiation therapy, and hormone therapy [14]. Progressive cancer and inoperable tumors require neoadjuvant chemotherapy, which aims to reduce tumor size [16,17]. Considering the complexity of tumors, treatment effectiveness requires tumor response assessment and adjustment using pharmacogenetic methods.

Treatment assessment is complicated by the application of combined chemotherapy regimens, which commonly include two or more anti-cancer drugs, or the administration of anti-cancer drugs in combination with hormonal therapy or immunotherapy [18]. Among the prescribed regimens are cyclophosphamide and anthracycline drugs, taxanes, and platinum-based drugs. According to clinical guidelines, one of the components of BC chemotherapy regimens is doxorubicin (Dox), which has been the standard anti-BC treatment agent for decades [16,17,18,19,20,21]. The pharmacokinetics of Dox (the processing of the drug by the organism) are very diverse and depend on the genetic profile of proteins responsible for metabolism, transport, and repair of the drug and its metabolites [21]. The whole process is also complicated by pharmacodynamics (effects of the drug on the organism), because the drug may influence epigenetic regulation and force some genes to becomes silenced and others to become activated [22]. Therefore, personal variations (patient genotype), such as single nucleotide polymorphisms (SNPs) in the enzyme structure, are responsible for the efficacy and toxicities of anti-cancer agents, leading to a personalized medicine approach for BC treatment. The orchestrated response to anti-BC therapies is described by pharmacogenetics, which questions the role of personal DNA in chemotherapy effectiveness [22]. Notably, phase I activations, phase II detoxification enzymes, and drug transmembrane carriers (including ATP-binding cassette (ABC) transporters) were shown to define Dox pharmacokinetics [21,22,23,24]. This study considers the association between gene polymorphisms and Dox-induced effects in BC patients. Associations between the expression of variants of Dox-metabolizing enzymes and successful BC treatment outcome are also discussed.

## 2. Pharmacogenetics of Dox Metabolism

**Figure 1:**
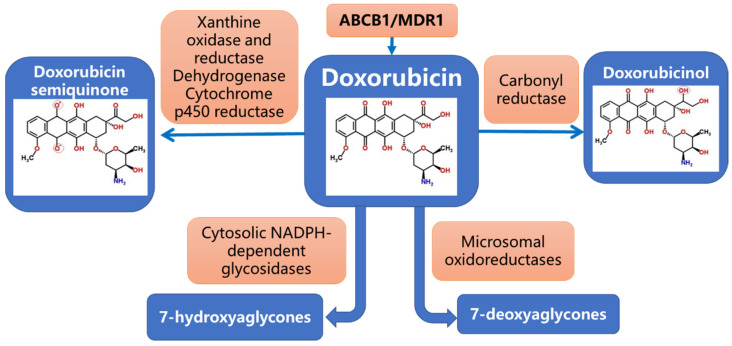
Major enzymes and products of doxorubicin metabolism pathway.

The metabolic transformation of Dox may follow several pathways, including two-electron reduction with the formation of doxorubicinol, one-electron reduction with the formation of semiquinone, and deglycosylation with the formation of aglycone. Several enzymes have been shown to be involved in this process (Figure 1) [24,25,26,27]. Doxorubicinol is considered the most dangerous metabolite of Dox degradation, as it may disturb iron and calcium balances [24,27].

Cytochrome P450 enzymes, including CYP3A4 and CYP2D6 (both enzymes are constitutively expressed in adult hepatocytes) and p-glycoprotein (mainly expressed in the liver, gastrointestinal (GI) tissues, kidney, and blood–brain barrier (BBB)) are the proteins which control Dox metabolism [26,27]. Dox is a substrate for CYP3A4/CYP2D6 and p-glycoproteins [28] which processes and/or transports this drug. Polymorphisms of the genes, encoding Dox-metabolizing enzymes, direct the outcome of this transformation and efficacy of the treatment [29]. The relationship between CYP3A4, CYP2D6, and p-glycoprotein gene polymorphisms, efficacy of the anti-cancer treatment, and development of adverse reactions to Dox are discussed below.

### 2.1. CYP3A4 Polymorphism

CYP3A4*1B is one of the most studied polymorphisms of CYP3A4 in cancer patients. Current data on the enzyme activity and its impact on the chemotherapy effects are conflicting. Tavira et al. (2013) demonstrated an association between the expression of CYP3A4*1B variants and increasing drug concentration in blood serum [30]. However, other studies reported a minimal influence of CYP3A4*1B on drug concentration [31]. This contradicts what was previously thought to be the role of this enzyme in the Dox conversion. Decreased metabolic activity of CYP3A4 may be caused by the presence of the CYP3A4*22 polymorphism. Several studies have reported that expression of this gene variant leads to an increase in various drug concentrations [32]. Interestingly, a meta-analysis study reported that CYP3A4*22 is a wide-spread polymorphism among Europeans (58.8%) and admixed Americans (82.4%) [33]. The CYP3A4*15 polymorphism was also found in 73.8% of Africans, while CYP3A4*18 was found in 63.4% of East Asians [33]. The role of the CYP3A4*15 polymorphism has not yet been clarified. The CYP3A4*18 polymorphism resulted in decreased enzyme function [33,34].

Other genes, including X-pregnane receptor (PXR) polymorphism, were found associated with CYP3A4 expression and regulated responses to BC treatment [34]. The expression of PXR mRNA in liver tissues of patients carrying clusters of PXR*1B haplotypes was found to be four times lower than that in people with the non-PXR*1B haplotype (*1A + *1C) clusters [34]. The PXR*1B haplotype also correlated with significantly lower CYP3A4 (and p-glycoprotein ABCB1) expression in the liver. Notably, Dox clearance in BC patients with the PXR*1B haplotype was significantly lower compared to non-PXR*1B patients [34]. Expression of the PXR*1B haplotype correlated with a lower Dox clearance, suggesting prolonged circulation of the drug and its higher therapeutic effects in Asian BC patients [34]. However, the effect of the CYP3A4 polymorphism on the metabolism and effectiveness of Dox in different BC cohorts remains largely unclear and warrants further investigations.

### 2.2. CYP2D6 Polymorphism

The CYP2D6 gene is marked by a high allele heterogeneity which reflects abundant inter-individual variations. The gene variants were grouped according to levels of enzyme activity. The described association between CYP2D6 polymorphisms and enzyme activity is presented in Table 1 according to the previously reported analysis [33]. The difference in distribution of CYP2D6 alleles in various populations was assessed and reported [33]. The CYP2D6*2 allele (normal-function allele) was found expressed in 56.3% of admixed Americans, 49.3% of the South Asians, 51.3% of Europeans, 29.5% of Africans, and 16.2% of East Asians. The alleles CYP2D6*3 and CYP2D6*6 (no-function alleles) were found less expressed in Europeans (4% and 6%, respectively), while the CYP2D6*10 allele (decreased function) was found almost exclusively in Africans, East Asians, and South Asians. The CYP2D6*1xN and CYP2D6*2xN alleles (increased function) were found in Europeans, Africans, and East Asians at a low frequency of 1.2–3.6% [33]. Considering that Dox is a substrate of CYP2D6, the rate of Dox metabolism is expected to correlate with this enzyme’s activity: the higher the CYP2D6 activity, the less amount of Dox that remains in the circulation (reduced therapeutic effect). It has been estimated that about 50% of admixed Americans, Europeans, and South Asians are likely to have normal Dox metabolism [33,34], and should therefore respond well to Dox-based anti-cancer therapies. However, this suggestion requires evidence-based confirmation. A meta-analysis study conducted in 2013 did not confirm the reliability of CYP2D6 genotyping as a guideline marker for anti-BC therapies [35]. However, the included studies were analyzing the effects of tamoxifen, not Dox-treated patients [35]. BC heterogeneity, confounding pre-selection of suitable patients for the treatment with tamoxifen, and differences in enzyme activity with Dox and tamoxifen as substrates may explain the observed contradictions. Analysis of associations between expression of all CYP2D6 variants in BC patients from different ethnic groups, their responses to Dox, and types of BCs has not been reported. The absence of data indicates an urgent need to estimate the level of CYP2D6 polymorphism in BC cohorts and its specific correlation with Dox metabolism and its therapeutic effects.

### 2.3. P-Glycoprotein Polymorphism and Dox Blood Concentration and Clearance

**Figure 2:**
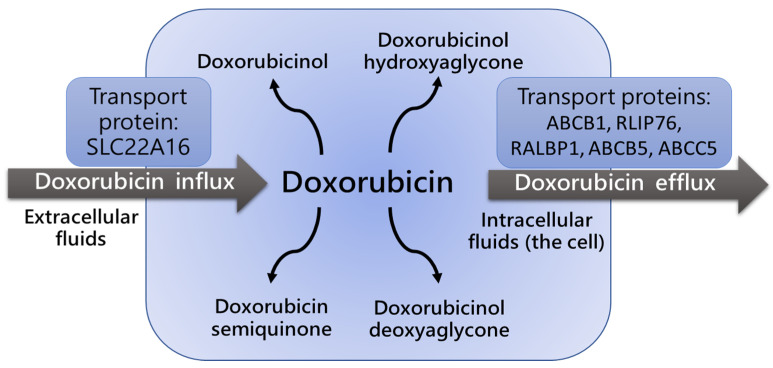
Influx and efflux of doxorubicin is defined by activity of ABCB1/MDR1 transport [39,40,41,42]. ABCB1/MDR1 protein expression level and polymorphism determine the intensity of doxorubicin transport.

P-glycoproteins, including ATP-binding cassette (ABC) family members such as ABCB1 transporters (also known as Multi-Drug Resistance 1 (MDR1) proteins), are responsible for Dox cell influx and efflux (Figure 2), and regulate both intra- and extracellular concentrations and bioavailability of the drug and its metabolites. A very limited number of studies estimated the impact of ABCB1 gene polymorphisms on Dox pharmacokinetics and pharmacodynamics [34,36,37,38,39,40], although the role of the ABCB1/MDR1 transporter in the regulation of intracellular concentration of anti-cancer agents and their therapeutic effects were reported [41,42]. The association between p-glycoprotein ABCB1 gene polymorphisms and changes in Dox concentration and clearance were reported [36,38,41,42]. The most studied variants are C3435T, C1236T, and G2677T/A. The distribution of allelic variation was associated with ethnicity. For instance, the 3435C>T variant was found in 60–72% of Asians and 34–42% of Europeans [36,37]. The distribution of ABCB1 haplotypes 1236C>T, 2677G/T, and 3435C>T was assessed in different races [38]. Among Africans, the wild-type (CGC) allele was found to be predominant, compared to the presence of the TTT allele. In Europeans, CGC and TTT allele frequencies were found expressed at similar levels. However, the TTT haplotype prevailed among Asians and Indians [38].

The role of C3435T polymorphism In the ABCB1 gene was recently investigated in patients with BC treated with Dox and docetaxel [36]. Patients with the C3435TT genotype had higher AUC and greater overall survival compared with patients with the CC⁄CT genotype. However, the TT genotype was also associated with higher risk of neutropenia and diarrhea. This genotype was found in 14.4% of the 216 enrolled patients [36]. It remains unclear which ABCB1 variants are linked to the most efficient effects of Dox in BC patients and which are associated with the poor survival outcomes and/or toxic effects of the drug.

A recent study indicated the influence of ABCB5, ABCC5, and RLIP76 polymorphisms on the pharmacokinetics of Dox in BC patients [40]. Genetic analysis was performed using direct sequencing. The homozygous variant allele at locus ABCC5g + 7161G4A (rs1533682) was significantly associated with higher Dox clearance [40]. Homozygosity of the reference allele at the ABCC5 locus g.-1679T4A was associated with significantly higher doxorubicinol blood concentration. No significant effect of ABCB5 polymorphisms (c.2T4C, c.343A4G, and c.1573G4A) on Dox pharmacokinetics was identified. RLIP76 gene polymorphisms were not reported. Therefore, Dox pharmacokinetics and pharmacodynamics may be influenced by ABCC5 gene polymorphisms [40]. However, the role of tissue specificity in the expression of this variant remains to be determined. It is necessary to confirm the metabolic transformation of Dox and the enzyme activity in the liver as a requirement for the effective retention of Dox in circulation.

## 3. Genetic Polymorphisms of Detoxifying Enzymes and Drug Resistance

Blood concentration of anti-cancer drugs correlates with tumor response. The accumulation of a drug at an effective dose can be altered by ABCB1/MDR1 p-glycoprotein functioning [43]. Concentration of the drug at less effective doses in circulation and/or in the cancer tissue may result in survival of cancer cells and development of drug resistance. It was demonstrated that MDR1 and glutathione S-transferase (GST) genes are involved in Dox resistance [44]. GST is the detoxifying enzyme which defines the sensitivity of cells to anti-cancer (toxic) chemicals [45]. Genetic polymorphism of both MDR1 and GST genes was associated with limited responses to chemotherapy [45,46,47]. Accordingly, BC recurrence and mortality rate were lower among patients with homozygous deletions of GSTM1*0 and GSTT1*0 compared to patients with the wild-type genotype, indicating the important role of detoxifying enzymes for the therapy responses.

The association between single nucleotide polymorphisms of the ABCB1/MDR1 gene and alterations in Dox and daunorubicin metabolism were also reported [48]. Higher rates of drug resistance were observed in carriers of MDR1 SNPs M89T, L662R, R669C, and S1141T. Alternatively, the presence of W1108R resulted in lower chemotherapy resistance [48]. Conflicting data about the role of MDR1 3435C>T were reported, demonstrating that there is an association between the MDR1 TT genotype and a worse tumor response to chemotherapy [49]. A recent meta-analysis study tested associations between chemotherapy response and the presence of C3435T, C1236T, and G2677T/A MDR1 polymorphisms [50]. Surprisingly, no significant association between ABCB1/MDR1 polymorphisms and response to chemotherapy was found in every genetic model assessed in this study [50].

Another recent study also investigated the association between Multi-Drug Resistance protein 2 (MRP2) (known as ABCC2, another member of ABC transporter family) gene polymorphisms and chemotherapy response [51]. The study assessed 181 patients with advanced BC and detected 226 SNPs in 15 genes. A significant association was found between response to Dox therapy and the rs717620 polymorphism of the ABCC2 gene. The presence of this gene variant resulted in the reduced effectiveness of Dox [51]. The possibility to use these variants as a potential biomarker for prediction of treatment outcome requires further validation in BC patients.

## 4. Genetic Polymorphisms and Cardiotoxicity in Dox-Treated Patients

Cardiotoxicity is one of the common adverse effects of anthracycline treatment [52,53,54,55,56] and the main limiting factor of this anti-cancer therapy. Although the pathophysiology of anthracycline-induced cardiotoxicity (ACT) is not fully established [57], ACT intensity depends on a cumulative dose of the drug, which is defined according to a patient genotype and should be personalized [58]. Redox cycling of Dox includes an interaction of the formed semiquinone compound with oxygen to produce the superoxide anion, reactive oxygen species (ROS) [59]. Dox-induced formation of ROS may result in the increased membrane lipid peroxidation of various organelles, such as mitochondria [60]. ROS formation is often registered during anthracycline drug (such as Dox) treatment, which triggers toxic cardiovascular (CVS) effects. ROS formation leads to DNA damage, cardiomyocytes apoptosis, ferroptosis [61], and inhibition of cellular protein synthesis [60]. A high number of mitochondria and low antioxidant defense of cardiomyocytes make these cells vulnerable to oxidative damage by ROS [62,63,64,65]. Dox-induced production of ROS leads to dysregulated calcium and iron transport [61,64,65] and reduced oxidative phosphorylation (respiration) and ATP production [64,65,66]. Dox was also shown to block the antioxidant system in cardiac muscles, represented by the sirtuins family proteins SIRT1 and SIRT3 [67,68]. DNA damage and higher expression of topoisomerase IIβ promoted cardiotoxicity during chemotherapy [69]. Accordingly, deletion of the topoisomerase IIβ gene resulted in cardioprotective effect in response to anthracyclines-induced DNA damage and reduced ROS production [59,64,65,69]. Population-based data indicated dose-dependent CVS toxicity of Dox in the vulnerable patients. The European Society of Cardiology supported the collection and analysis of data regarding the occurrence of left ventricular dysfunction detected after Dox therapy [69,70,71]. The incidence of Dox-linked adverse effects was found growing along the increases in cumulative Dox doses [70,71,72]. Therefore, cumulative doses of Dox should be carefully estimated in vulnerable groups of patients with high risk of CVS toxicity.

Cardiomyocyte-protecting protein variants were also found involved in Dox-linked toxicity. The expression of SNPs in CUGBP (RNA-binding protein) and ELAV-like family member 4 (CELF4, involved in regulation of mRNA metabolism) was evaluated in association with cardiotoxicity in children [73]. Interestingly, the rs1786814 genotype of CELF4 was found associated with expression of the cardiac troponin T (TNT)-encoding gene (TNNT2) in cardiomyocytes and cardiomyopathy. The ventricular contractility reduction was found associated with the polymorphism in CELF4 [73].

The role of ABC transporters (including MDR1) in the Dox-induced cardiotoxicity was investigated and reported. Polymorphisms A1629T in the ABCC5 gene and G894T in the endothelial nitric oxide synthase 3 (NOS3) gene were reported to influence the development of cardiotoxicity in children [74]. Patients with the ABCC5 TT-1629 genotype had a reduced left ventricular ejection fraction by 8–12% [73]. Another group demonstrated that acute ACT was associated with the expression of the Gly671Val variant of MRP1 and with the Val1188Glu-Cys1515Tyr (rs8187694-rs8187710) haplotype of the MRP2 (Dox efflux transporter) [75]. Furthermore, the expression of A-1629T, rs7627754 (ABCC5 gene), rs4148808 (ABCB4 gene), and the homozygous G allele of carbonyl reductase 3 (CBR3) gene were associated with cardiotoxicity in children [76].

Aside from ABC transporters (ABCC1, ABCC2, ABCC5, ABCB1, ABCB4), genetic variants of NOS3 [74], CBR3 [76,77], cytochrome B-245 alpha chain (CYBA) [78], GST protein 1 (GSTP1) [79], hydroxysteroid sulfotransferase 2B1 (SULT2B1) [80], p450 oxidoreductase (POR) [81], organic anion transporters (solute carrier family 22 members 7 and 17 (SLC22A7 and SCL22A17) and SLC family 8 member 3 (SLC28A3)) [80], iron-metabolism-regulating protein (human hemochromatosis (HFE)) [82], and retinoic acid receptor-gamma (RARG) [57] were associated with ACT. However, the physiological and molecular links between the indicated genes and development of ACT require additional validation in population-based studies. The systematic review of the available data demonstrated that RARG variant rs2229774, SLC28A3 variant rs7853758, and UDP-glucuronosyltransferase 1-6 (UGT1A6) gene variant rs17863783 correlated with the incidence of ACT [57]. The expression of the UGT1A6*4 variant was linked to the decreased enzyme activity, which resulted in the decreased rate of Dox metabolism [76]. Interestingly, the RARG variant effect was found associated with the inhibition of topoisomerase IIβ expression, indicting the link to DNA damage in cardiomyocytes [83]. The expression of RARG rs2229774 (S427L), SLC28A3 rs7853758 (L461L), and UGT1A6*4 rs17863783 (V209V) variants was also found associated with therapeutic responses to anthracyclines in BC patients [84], although further pharmacogenetic testing in larger cohorts is recommended.

Toxicity-linked pharmacogenetics of the cytochromes P450 have been reported. Bray et al. (2010) studied the influence of SNPs in ABCB1 (C1236T, G2677T/A, and C3435T), SLC22A16 (A146G, T312C, T755C, and T1226C), CYP2B6 (-*2, *8, *9, *3, *4, and *5), CYP2C9 (-*2 and *3), CYP3A5*3, and CYP2C19*2 on chemotherapy-induced cardiotoxicity in 230 BC patients [85]. The study discovered that carriers of SLC22A16 A146G, T312C, and T755C variants had lower levels of cardiotoxicity. Alternatively, higher toxicity was found in patients with SLC22A16 1226C, CYP2B6*2, and CYP2B6*5 alleles [85]. However, the involvement of different p450 gene variants in drug-induced cardiotoxicity remains unclear. The role of p450 cytochrome polymorphism in Dox-induced cardiotoxicity warrants further population and genome-wide investigations. It is essential to define the plausible therapeutic targets which can be used to reduce CVS-linked Dox toxicity. Detoxifying and oxidative-stress-reducing enzymes represent promising candidates for this purpose [86].

## 5. Genetic Polymorphisms Associated with Dox-Induced Hematological, Nephrological, and Gastrointestinal (GI) Toxicities

### 5.1. Role of Gene Polymorphism in Dox-Associated Hematotoxicity

ABC transporter gene polymorphism was reported to influence drug-induced CVS toxicity. The association between chemotherapy-induced neutropenia and ABCB1 polymorphism was evaluated in 141 BC patients treated with Dox and cyclophosphamide [87]. Effects of ABCB1 gene polymorphisms (2677G>T/A and 3435C>T) were estimated using multivariate logistic regression analysis. Data showed that polymorphism 2677G>T/A may be used to predict neutropenia [87]. The assessment of a link between myelosuppression and other ABCB1 polymorphisms (C1236T and C3435T) was conducted in a study with 72 BC patients [88]. The frequencies of the CC, CT, and TT genotypes of the ABCB1 C1236T gene were 11 (15.28%), 42 (58.33%), and 19 (26.39%), respectively. However, no significant associations were found between ABCB1 (C1236T and C3435T) polymorphisms and myelosuppression (*p* > 0.05) [88]. A larger study by Yao et al. (2014) which included 882 patients with BCs showed that SNPs in ABCC1 (809 + 54C> A (rs903880), 677 + 1391T> C (rs16967126), and 1988 + 219G> T (rs4148350)) served as good predictors of hematotoxicity [89].

A more recent study by Tecza et al. (2018) evaluated the genetic and clinical risk factors of anthracycline-induced toxicities in 324 BC patients [23]. The study assessed the polymorphism of selected genes involved in drug transport (ABCB1, ABCC2, ABCG2, and SLC22A16), metabolism (ALDH3A1, CBR1, CYP1B1, CYP2C19, DPYD, GSTM1, GSTP1, GSTT1, MTHFR, and TYMS), DNA damage recognition, repair, and cell cycle control (ATM, ERCC1, ERCC2, TP53, and XRCC1). Multivariate logistic regression analysis detected a correlation between genetic or clinical factors and the manifestation of anemia, leukopenia, and neutropenia. For instance, the risk of chemotherapy-induced anemia correlated with the polymorphic allele p.Asn118 = (rs11615) in the ERCC1 gene and homozygous GG polymorphism p.Val417Ile (rs2273697) of the ABCC2 gene in triple-negative BC patients [23]. The presence of a rare G allele of the p.Pro329Ala variant in the ALDH3A1 gene and homozygous CC polymorphism of the ABCB1 gene p.Ile1145 = (rs1045642) led to the recurrence of anemia. Variations in ABCG2 were also associated with early anemia. The presence of heterozygote CA of the p.Gln141Lys (rs2231142) variant was associated with increased risk of early anemia [23]. Multivariate logistic regression analysis revealed that allele G of p.Pro329Ala in the ALDH3A1 gene (rs2228100) and CYP2C19 c.-806C>A (rs12248560) common homozygote CC increased the risk of leukopenia [23]. Furthermore, severe neutropenia was associated with independent genetic factors, including expression of the 3R3R variant of TYMS 28bp tandem repeat (rs34743033). The expression of the homozygote variant TT of ABCC2− p.Ile1324 = (rs3740066) and AA homozygote of DPYD p.Ile543Val (rs1801159) also elevated the risk of severe neutropenia [23].

To estimate the effects of polymorphism, Chen et al. (2016) conducted a meta-analysis and explored the impact of CBR1, ABCB1, ABCC1, ABCC2, ABCG2, and SLC22A16 genes on Dox-induced toxicity [90]. The study reported that the presence of the T allele of the ABCB1 2677G>T/A gene was associated with a higher number of platelets in a blood sample, while carriers of the ABCB1 IVS26 + 59 T>G gene (rs2235047) had higher levels of neutrophils and leukocytes. The ABCC2 3563T>A (rs8187694) and 4544G>A (rs8187710) gene polymorphisms significantly correlated with the risk of Dox-induced ACT, although the association did not remain significant after adjusting for age, gender, cumulative Dox dose, and dosing interval [90]. To prevent or reduce cardiotoxicity during chemotherapy regimens, SNP/genetic variants responsible for increased CVS toxicity should be identified, confirmed, and used as markers for the choice of anti-cancer drugs.

### 5.2. Role of Gene Polymorphism in Dox-Associated Nephrotoxicity and GI Toxicity

Several genetic factors involved in the regulation of Dox-induced nephrotoxicity and GI toxicity were detected. Expression of the polymorphic allele C of endonuclease ERCC1 gene variant p.Asn118 = (rs11615) was associated with increased risk of kidney damage [21,23]. No other gene polymorphisms associated with adverse effects in the kidney were reported in BC patients after Dox treatment. Nephrotoxicity-related roles of different gene variants which encode important detoxifying and transporting enzymes warrant future investigations.

The homozygote CC of the CYP1B1 p.Leu432Val variant was defined as the most frequent predictor of Dox-induced GI toxicity [23]. Expressions of the rare allele A of ATM p.Asp1853Asn (rs1801516) and the common allele A of GSTP1 p.Ile105Val (rs1695) were shown to increase nausea risk, although the effect was found to be significant at the threshold level [23]. The summary of the reported candidate genes and polymorphisms associated with Dox adverse health reactions is presented in Table 2.

## 6. Future Perspectives

The rate of gene modifications in the population (also defined as gene penetrance) is one the major indicators of gene polymorphism level in BC patients. For instance, BRCA1 and BRCA2 genes have higher penetrance rates compared to TP53, PTEN, and SKT11 (LKB1) in BCs [91]. However, CYP450 and p-glycoprotein polymorphism penetrance remains poorly investigated. Furthermore, a recent meta-analysis revealed no association between ABCB1/MDR1 polymorphisms and responses to chemotherapy [50], although other studies found the relations between MDR1 polymorphisms and drug resistance rate [44,48,49,51]. The difference between these findings may be associated with population-based variations in gene penetrance (phenotype presentation rate). Notably, environmental factors and epigenetics play a significant role in phenotype presentation. The penetrance of the CYP450/ABCB1 polymorphism can be established only through large population-based studies.

Epigenetic mechanisms of gene regulation, including DNA methylation, histone modification, and non-coding RNAs (such as microRNA (miRs)) are involved in the regulation of gene expression [92,93,94,95,96,97], and therefore can be potentially targeted in BC patients with CYP450/MDR1 polymorphisms. It has been demonstrated that miR-1, miR-208, and miR-133 are associated with anthracycline cardiotoxicity [97]. The use of epigenetic modulators along with chemotherapy has been recommended to overcome drug resistance [96,98]. Upregulation of miRs responsible for control over MDR1 expression was also observed [97]. It has been found that miR-451 and miR-27a caused an increased level of MDR1 in neoplastic cells [97,99]. Similar data were reported for miR-298 [100], let-7b [101], and other miRs in drug-resistant BCs [102,103,104]. However, it remains unclear whether the abovementioned miRs can provide an effective regulation of expression of CYP450 gene variants. Moreover, future studies should confirm whether the application of epigenetic modulators is equally effective with different variants of the same gene. Currently tested effects of non-coding miRs were reported without confirmation of genetic differences.

The combined application of Dox with epigenetic drugs, either as mixed solutions or encapsulated agents in nanogels, was found to be more efficient than the drug alone [96]. The use of nanocarriers (nanoparticles (NPs)), including liposomes, polymers, electro-sprayed particles, and nanosuspensions, was suggested as a promising approach to minimize adverse side effects of Dox [19,20,102,105,106,107,108,109]. Several types of NPs were found to improve the pharmacokinetic characteristics of anti-cancer agents and provide better targeted delivery and controlled release into cancer cells [19,105,106,107]. Improved pharmacokinetic parameters were demonstrated for liposome-incorporated Dox [19,106]. Delivery of Dox by nanocarriers extended the drug plasma half-life and slowed its clearance without increases in gastrointestinal toxicity and cardiotoxicity [110]. Non-pegylated and pegylated liposomal Dox forms were approved for clinical treatment [20,105,106,107,108,109]. Application of nanotechnology may provide a solution for those patients with genetic polymorphism in CYP450 and/or MDR1 genes, although the degree of success with NP-loaded Dox/miRs remains to be assessed. The employment of nanocarriers for Dox delivery, as a method to improve Dox pharmacokinetics and reduce drug resistance, warrants future investigations.

## 7. Conclusions

Targeted BC treatment is complicated by cancer heterogeneity, which is represented by the expression of different sets of cancer-regulating genes and gene variants, defined as gene polymorphism [111,112]. A personalized medicine approach is designed to address the complexity of cancer treatment and involves a combination of different methods and drugs targeting several cancer cell death activating effectors and pathways. However, gene polymorphism impacts drug response at many levels, including the drug metabolism and downstream biological responses to chemotherapy. Consequently, carriers of specific gene variants develop various adverse reactions to chemotherapy, ranging in severity. To minimize toxic side effects and optimize the cancer killing outcome, a personalized medicine approach requires consideration of pharmacokinetics and pharmacodynamics for each prescribed drug. Effective therapy should be also accompanied by careful monitoring of patient condition and timely therapy adjustments.

Dox is often prescribed for BC patients as part of a combined radio-chemotherapy approach [21,23,27]. Gene polymorphism strongly influences the effectiveness and safety of BC therapy, including drug retention and toxicity. Among the enzymes responsible for Dox metabolism and cell transport are ABC transporters (MDR proteins), p450 cytochromes, and other detoxifying enzymes [28,35,36,37,38,39,40]. However, polymorphism of these genes and its role in the regulation of Dox responses remain under-addressed. The prognostic value and effects of gene polymorphism of Dox-metabolizing enzymes (including ABCC and CYP1B1 polymorphism) on BC survival were not reported, although large BC databases have been made publicly available for some time. The prognostic analysis may be completed using, for instance, the Kaplan–Meier Plotter database (https://kmplot.com/analysis, accessed on 30 September 2022). Personalized medicine approaches cannot be designed without an understanding of individual pharmacogenetic characteristics that can reflect altered Dox pharmacokinetics and change the blood concentration of the drug. The treatment efficacy cannot be predicted without a clear understanding how Dox will be metabolized and how quickly it will be cleared by the carrier of specific gene variants. Some progress has been made towards the discovery of expression and functional roles of ABC transporters and p450 cytochrome gene variants in BC patients. However, it remains to be confirmed which set of gene variants defines Dox pharmacokinetics/dynamics. This review focused on the reported candidate genes involved in Dox metabolism, efficacy, and safety. The main set of gene candidates includes P-glycoprotein genotype variants (ABC drug transporters/MDR proteins) and cytochromes (CYP), which were also associated with Dox-induced toxicities (summarized in Table 2). The observed adverse effects of Dox may be diminished using epigenetic and nanotechnology methods of cancer-cell-targeted drug delivery [94,95,113,114].

A handful of research studies assessed MDR1 polymorphisms in BC patients and their role in Dox effects. Expression of gene variants for CYP3A4 and CYP2D6 proteins have been studied, although the data require confirmation in a larger BC cohort. The associations between incidence and severity of Dox adverse reactions and CYP3A4 and CYP2D6 polymorphisms remains unclear. It is also unclear how pro-inflammatory conditions, including immunotherapies and low level of inflammation in obese patients [115,116], will impact the Dox pharmacokinetics and therapeutic effectiveness [117]. Future clinical genome-wide studies should define and confirm the set of gene variants which influence Dox safety and efficacy.

## Figures and Tables

**Table 1:** CYP2D6 polymorphisms and the enzyme activity [33,34,35].

No-Function Alleles	Decreased-Function Alleles	Normal-Function Alleles	Increased-Function Alleles
*3, *4, *4xN, *5, *6, *7, *8, *11, *12, *36, *40, *42, and *56	*9, *10, *17, *29, *41, *44, and *49	*2, *35, *43, and *45	*1xN, *2xN

**Table 2:** Candidate genes and polymorphisms associated with Dox-induced adverse reactions.

Dox-Related Effects	Gene/Polymorphism	Reference
Drug clearance	ABCC5g + 7161G4A (rs1533682) expression resulted in faster Dox clearance; homozygote allele in ABCC5 g.-1679T4A locus was linked to higher Dox concentration in blood.	Lal et al. (2017) [40]
	PXR*1B haplotype was linked to lower Dox clearance.	Sandanaraj et al. (2008) [34]
	C3435TT genotype was associated with higher AUC.	Kim et al. (2015) [36]
Drug resistance	GSTM1*0 и GSTT1*0 presence was associated with lower risk of disease relapse/death.	Stearns et al. (2004) [44]
	Drug resistance was detected in carriers of M89T, L662R, R669C, and S1141T polymorphisms of ABCB1 gene; lower level of drug resistance was shown in carriers of ABCB1/W1108R variant.	Jeong et al. (2007) [48]
	MDR1 TT genotype was associated with a worse tumor response to chemotherapy.	Tulsyan et al. (2016) [49]
	No association was found between ABCB1/MDR1 polymorphisms and response to chemotherapy (meta-analysis study).	Madrid-Paredes et al. (2017) [50]
	Lower Dox efficacy was associated with expression of ABCC2/rs717620 variant.	Ruiz-Pinto et al. (2018) [51]
Cardiotoxicity	Increased risk of toxicity was associated with A1629T in ABCC5 and G894T in NOS3 genes.	Krajinovic et al. (2016) [74]
	Expression levels of Gly671Val/MRP1 and Val1188Glu-Cys1515Tyr (rs8187694-rs8187710)/MRP2 variants were linked to the increased risk of acute cardiotoxicity.	Wojnowski et al. (2005) [75]
	RARG variants rs2229774, SLC28A3 rs7853758, and UGT1A6 rs17863783 correlated with the increased toxicity.	Aminkeng et al. (2016) [57]
	ABCC5 (A-1629T, rs7627754) and ABCB4 (rs4148808) correlated with the decreased left ventricular ejection fraction.	Armenian et al. (2018) [76]
	Expression of rs1786814/CELF4 gene was associated with the decreased myocardial contractility.	Wang et al. (2016) [73]
	SLC22A16 variants A146G, T312C, and T755C correlated with the lower toxicity, while SLC22A16 variants 1226C, CYP2B6*2, and CYP2B6*5 were linked to the higher toxicity.	Bray et al. (2010) [85]
Hematotoxicity	Expression of ABCB1 variant 2677G>T/A was linked to the higher risk of neutropenia.	Ikeda et al. (2015) [87]
	No significant associations were found between ABCB1 (C1236T and C3435T) polymorphisms and myelosuppression.	Syarifah et al. (2018) [88]
	ABCC1 variants 809 + 54C>A (rs903880), 677 + 1391T>C (rs16967126), and 1988 + 219G>T (rs4148350) correlated with the higher toxicity.	Yao et al. (2014) [89]
	Allele p.Asn118 = (rs11615) in gene ERCC1, homozygote GG polymorphism p.Val417Ile (rs2273697) of ABCC2 gene, and heterozygote allele CA of variant p.Gln141Lys (rs2231142) correlated with the higher risk of anemia. G allele of p.Pro329Ala in gene ALDH3A1 (rs2228100) and homozygote CC allele of CYP2C19 c.-806C>A (rs12248560) were linked to the higher risk of leucopenia. TT allele of ABCC2 gene was associated with the higher risk of severe neutropenia.	Tecza et al. (2018) [23]
GI- and nephrotoxicity	Homozygote allele CC of gene polymorphism CYP1B1, A allele of gene ATM p.Asp1853Asn (rs1801516), and A allele of gene GSTP1 p.Ile105Val were linked to the higher risk of toxicity.	Tecza et al. (2018) [23]
	C allele of gene ERCC1 variant p.Asn118 = (rs11615) and expression of GSTT1 and GSTM1 genes was associated with the higher risk of toxicity	
